# Factors Associated with Fatality in Ontario Thoroughbred Racehorses: 2003–2015

**DOI:** 10.3390/ani11102950

**Published:** 2021-10-13

**Authors:** Peter Physick-Sheard, Amanda Avison, William Sears

**Affiliations:** 1 Department of Population Medicine, University of Guelph, Guelph, ON N1G 2W1, Canada; wsears@uoguelph.ca; 2 DVM Program, Ontario Veterinary College, University of Guelph, Guelph, ON N1G 2W1, Canada; aavison@vet.upenn.edu

**Keywords:** sustainability, training, racing industry, work intensity, equine welfare, social license, risk factors, sudden death, musculoskeletal injury

## Abstract

**Simple Summary:**

Ontario’s Alcohol and Gaming Commission records equine racing fatalities through its Equine Health Program. This study examines the 695 Thoroughbred occurrences from 2003 to 2015 with the aim of identifying associations. Deaths occurred within 60 days of a horse racing or being entered or qualified to race. Calculated on the most commonly adopted basis, rates for Ontario Thoroughbreds are high—2.94/1000 starts (all fatalities) and 1.96/1000 (breakdowns only), compared with a global industry breakdown range of 0.29–2.36/1000 starts. The study revealed several significant risk factors, including workload, stage of training, age, sex, stage of career, finish position, race field size, and day of week. Among fatalities were groups where combinations of these risk factors were important, such as being a two-year-old male, un-castrated, and in early training. Probability of fatality fell over the study period in response to increasing awareness through existence of the Program and adoption of new regulations, but remains of concern. All identified associations represent aspects of management and industry structure that are amenable to change with a view to reducing fatalities. A link between fatality and cumulative, non-fatal outcomes should also be considered. An overall approach might view associated factors as sources of physical and psychological stress that, acutely and cumulatively, may influence the liability for adverse outcomes in training and racing.

**Abstract:**

Ontario’s Alcohol and Gaming Commission records equine racing fatalities through its Equine Health Program. The present study examined all Thoroughbred fatalities from 2003 to 2015, inclusive, to identify associations. Official records and details of fatalities were combined in multivariable logistic regression modelling of 236,386 race work-events (433 fatalities), and 459,013 workout work-events (252 fatalities). Fatality rates were 2.94/1000 race starts (all fatalities) and 1.96/1000 (breakdowns only) with an overall rate of 2.61% or 26.1 fatalities/1000 horses. Comparison with published reports reveals rates to be high. Musculoskeletal injury was the predominant complaint and there was a high incidence of horses dying suddenly. Liability was high for young horses early in the season with a differential according to sex and whether a male horse was gelded. Horses undertaking repeated workouts had a higher liability and liability was higher in workouts for horses switching from dirt/synthetic to turf racing and for young horses in sprints. Race distance was not significant but high fatality rates in some large field, distance races combined with effects of age and workload identified groups at particular risk. As field size increased, fatality liability increased for early-finishing horses. Findings suggest jockey strategy could be an important factor influencing fatalities. Probability of fatality declined over the study period. Findings indicate that rapid accumulation of workload in animals early in their preparation is likely to be damaging. Fatality fell toward the end of a season and for horses with a long career history of successful performance; however, horses not exhibiting this robustness and staying power represent the population of greatest concern. Associations may be characterised as representing sources of stress, current or cumulative, and identifying at-risk animals on this basis may be as productive as targeting specific, discrete mechanisms suspected to contribute to individual fatalities.

## 1. Introduction

Injuries and fatalities in horse racing are issues for the general public [1,2,3] as well as for the industry [4], and have the potential to damage the industry’s social license to operate because of the associated welfare concerns. These issues have led to extensive research to identify underlying causes [5,6,7,8]. Morbidity and fatality are often approached in epidemiologic studies by enumerating specific presenting complaints, for example, musculoskeletal injuries (MSI) and breakdowns. Associations are then examined between horse, work, and racing environment factors and these adverse outcomes to gain insights into causation and identify preventive strategies. This approach has been particularly useful for MSI, the greatest source of loss [7,9,10,11,12,13], and many risk factors have been identified. Among other discoveries, it has led to recognition of the importance of bone strength and density and the manner in which this accrues, the importance of micro fractures, accumulated strain and the time required for adequate healing or adaptation to take place, and the overall impact of cumulative wear and tear [13,14,15,16,17].

Among factors associated with injury, sex has been found by some studies to be influential [9,18,19,20,21,22,23,24,25,26,27]. A positive association with increasing age has also been identified in some investigations [9,28,29,30,31,32,33,34,35,36,37]. Both have been associated with differing patterns and rates of injury or fatality, individually and through sex*age interactions. Patterns of work and training, rest periods, and workloads leading up to an injury have also been identified as significant [13,21,38,39,40,41,42]. Track quality has received consideration as a factor [43,44,45,46,47], as have trainer and training [48,49,50,51,52]. Other factors such as shoeing [21,53,54], pre-existing injuries [31,55,56], field size [19,30], and type of race [21,49,57] have also been incriminated.

The resulting picture is complex, and results do not always agree, and are sometimes contradictory [7,8,13,21,39,58]. Differences may reflect population and jurisdiction, inclusion/exclusion criteria, and definitions, while studies may vary in their success in identifying and controlling for explanatory variables. Investigations differ in their sampling frame and unit of interest and may consider problems on a single or on multiple tracks, only races or races plus training/qualifying injuries, or focus on only one type of presenting complaint. It is rarely possible to align findings of focused studies with overall management or general background attrition so as to place losses in the broad context of industry and industry practices. This results in contributing factors being missed or de-emphasised. Intense focus on one complaint such as MSI may also obscure the broad industry background within which losses occur.

The Ontario, Canada, provincial regulator of racing (the Alcohol and Gaming Commission of Ontario, AGCO) maintains a database of equine fatalities under its Equine Incidents in Ontario Racing (EIOR) program, a part of its Equine Health Program, which addresses all horse racing in the province, regardless of breed. Provincial Rules of Racing mandate that owners, trainers, and treating veterinarians provide written notification to the regulator within 2 days if a horse dies within 60 days of being entered or qualified to race. Penalties apply for failure to comply. Postmortem examination is mandatory where death takes place within 14 days and is otherwise at the discretion of the regulator. Horses withdrawn from a race (scratched) are captured by the program. The database thus covers all 60-day fatalities regardless of cause. These data provide an opportunity to gain insight into industry-specific background factors associated with fatality of all causes.

Previous descriptive analysis of these data for 2003–2015 revealed fatality patterns to vary according to breed-specific characteristics such as age, sex, stage of career, and competitive activity [27]. Thoroughbreds had the greatest exercise-associated fatality rate and risk by all measures (2.27 deaths/1000 events, 0.95–1.0% annual individual risk), and the highest overall fatality. Exercise-associated fatalities primarily involved MSI, dying suddenly, and accidents. The parallels between fatalities and industry characteristics suggest possible independent influences of management, industry structure, and competitive activity, and further breed-specific analysis is thus warranted. Fatality may be the end result of cumulative damage [13,14,15,37,59,60], and in the present data MSI predominate as the leading presenting problem. Findings may thus be of relevance to future studies of morbidity.

The objective in the present study was to examine associations with fatality in Ontario Thoroughbred racing using available performance records. Cases drawn from the AGCO database were treated generically in multivariable analyses without differentiating by the presenting problem and results thus apply to fatalities in general. The influence of variables of interest was examined by performing modelling with the unit of interest being work-event (race or workout), and the outcome being fatality. To assess annual individual horse impacts, data were also analysed with horse-year as the unit of interest, allowing direct comparison between different racing industries and jurisdictions while providing information of interest to owners, trainers, and managers of facilities. Horse-year was defined as a single horse competing for a calendar year. This study will provide guidance in the design of strategic interventions aimed at reducing fatalities.

## 2. Materials and Methods

Fatality data from the province of Ontario’s database of equine fatalities for 2003–2015 inclusive were made available for the study by AGCO under a confidentiality agreement guaranteeing client anonymity. Ontario hosted 2 Thoroughbred tracks during the study period, designated here as Track 1 (premier) and Track 2 (regional). Presenting complaints for fatalities were gathered from Registry Submission Forms that are completed by the submitting agent (usually the trainer or trainer’s agent) [27], and in most instances represented a diagnosis made by the attending veterinarian. Complaints were consolidated into nine groups as previously described [27]. This process was aided by review of postmortem reports for those cases so handled (61.78%), but presenting complaints were not changed to definitive diagnosis for those cases. In describing these data, the term “dying suddenly” is used as a simple clinical description in preference to “sudden death” to avoid assumptions concerning exercise association, cause, or other circumstance.

Fatality data were supplemented by performance data obtained from Equibase (Equibase Company LLC, 821 Corporate Drive, Lexington, KY, USA) and describing details of officially recorded races and workouts, from 1 January 2003 to 31 December 2015, inclusive for all Thoroughbred horses competing in the province. Races and workouts are collectively referenced below as work-events. A database was then built with the unit of interest being work-event and contained all work-events for the study period, totaling 695,409 records. A second, horse-year, database in which the unit of interest was horse-year was built from this work-events database and contained 44,639 horse-race-years and 52,658 horse-workout-years. For this second database, the last race event and the last workout event for each unique horse name in each year were identified. Individual horses could thus appear up to twice in a year in the horse-year database. All variables in each horse’s last workout and race record for each year were retained, so that summary work indices (see below) were the total for each horse for that year. In constructing the horse-year database, the cumulative number of races and of workouts for each horse for each year were determined and stored separately in the variable RWYN. Records in this horse-year database were modelled as being race or workout, separately, and no attempt was made to model the two together.

Outcome (dependent variable) in all models was fatality (DR = 1, membership in the AGCO fatal events database) or DR = 0. Independent variables used in multivariable modelling are presented in Table 1, which shows definition, range, and data type for each. Three sexes (female, stallion, and gelding) were identified and for each event sex was that recorded for that work-event. Trackside terms “filly”, meaning young female, “colt”, meaning young intact male, and “horse”, meaning mature stallion, were not used. Derived variables were race field size (RSIZE, number of starters in a race, see below), RWYN (number of races or workouts, as appropriate, for the current year, used only in modelling of horse-year data), and track surface (TS), a combination of TRACK and SURF used only in modelling of workouts, since workouts only occurred on one surface at Track 2 but on 3 at Track 1. The summary work indices CMCAR, CMD, CMYR, and RWYN were derived from race records, and with the exception of CMCAR, are annual statistics (Table 1). CMCAR was cumulative and included work-events from before 2003 for horses racing before the start of the study period (2003).

The work-event identified as associated with a fatality was the last work-event in which the horse participated prior to death. Available data did not allow the number of fatalities directly associated with training to be determined. Temporal characteristics of the association between work-event and fatality for these data have previously been explored [27]. Data analysis in this study was by calendar year. Reference to effects taking place within or over a calendar year is by use of the term “year”. The terms “racing season” or “season” refer specifically to that part of the year within which there is racing/race preparation at the tracks, usually late March to early December.

Frequency of fatality by race outcome (finish position) was examined (PROC FREQ, SAS 9.4a) and the continuous variable, finish position, was converted to the categorical variable FPOS by grouping finish positions with similar fatality rates. Groups were 2.5 (finish positions 1–4), 5.5 (positions 5 and 6), 9 (positions 7–11), and 12.5 (positions greater than 11). Horses failing to finish a race (variously described as “eased”, “pulled up”, “fell”, or “broke down”) are usually given last place finish positions in Thoroughbred racing, and reference must be made to full race charts to determine whether a horse actually completed the race. Because of varying field sizes, a horse could thus finish last in a range of numbered positions depending on field size. The number of starters or race field size (RSIZE) was therefore determined as the maximum finish position for each discrete race (race defined by track, race date, and race number) and added to records for each race for modelling as an independent variable.

### Statistical Analysis

Two sets of logistic regression analyses were performed using either PROC GENMOD or PROC GLIMMIX (SAS 9.4a) with a binomial response variable (outcome, DR, fatality or not) and logit link function. Choice of procedure depended on preferred output. In the first set of analyses, the unit of interest was work-event, and in the second, horse-year. Modelling proceeded by considering all main effects, then backward elimination was applied (preserving hierarchy). Terms taken out early were sequentially reintroduced to see whether they may have become significant after removing competing variables. Two-way interaction terms and quadratic (second order) effects were then introduced and examined. Stepwise addition and subtraction of terms was subsequently followed with retention of those significant at *p* < 0.05. Eventually, all variables and all possible two-way interactions, and for statistically significant continuous variables, second order effects, were considered, regardless of biological plausibility. Testing of three-way and four-way interactions was employed where indicated by combinations of significant two-way interactions. During construction of each model, model strength was assessed by monitoring type III tests of fixed effects (*F*-test *p*-value) to confirm significance of each term retained in the model. Prior univariable screening was not employed [61]. Contrast estimates were constructed using PROC GLIMMIX (SAS 9.4a). Significance was set at *p* ≤ 0.05. Because of the number of horses and records involved in the study, horse was not entered as a random variable in any model as this would have consumed all available degrees of freedom [62].

Variables CMD, CMYR, and CMCAR were divided by 10 and variable YD was divided by 30 in models to retain precision in significant estimates and to put results on a meaningful scale for biological interpretation. Estimates and odds were adjusted accordingly. Estimates used in constructing contrasts were obtained by holding continuous variables at their mean and categorical variables at their referent levels. Results are expressed as probabilities or odds, and as odds ratios (OR) where comparisons are made and are stated with their 95% confidence intervals and the significance level for the model estimate from which they were derived. For interaction terms, estimates actually provide ratios of odds ratios since the difference between two odds ratios is being generated. In describing models in the tables, intercepts, estimates, standard errors, approximate 95% confidence intervals (CI), and *p*-values are presented for significant and involved main effects. Odds ratios are presented together with their confidence intervals where those intervals would provide a meaningful representation of population variation. Results derived from the data outside of multivariable modelling are presented by reference to “raw” data. This study defines a horse finishing first in a race as having a high finish position and one finishing last as having a low position.

## 3. Results

The fatal incident database contained 695 Thoroughbred fatalities, with an age range from 2 to 10 years, with 237 geldings, 166 stallions, and 292 females. A total of 30 different terms were used to describe presenting problems on submission sheets and were consolidated into 9 categories (Table 2). MSI predominated, followed by dying suddenly and colic. Ten horses died in early training without participating in a race or workout and were not used in event-based multivariable analysis, leaving 685. The sex distribution of these 10 horses did not differ from the rest of population, but all were less than 5 years of age. Presenting problems for these horses were MSI (5) and accident (5).

Overall, raw fatality rates were 1.8317 and 0.5708 fatalities per thousand events for races and workouts, respectively (0.9994/1000 combined, Table 3). Fatality rate if all incidents were included but only race starts were considered in the denominator was 2.9401/1000 race starts. The total number of unique horses who participated in racing in Ontario during the study period was 26,625 for an absolute horse fatality rate of 2.61% or 26.1 fatalities/1000 horses. 

### 3.1. Modelling by Work-Event

Results of multivariable logistic regression modelling of associations with fatality by race work-event are presented in Table 4, and by workout work-event in Table 5. Modelling of all events combined is presented in Table S1.

**Figure 1:**
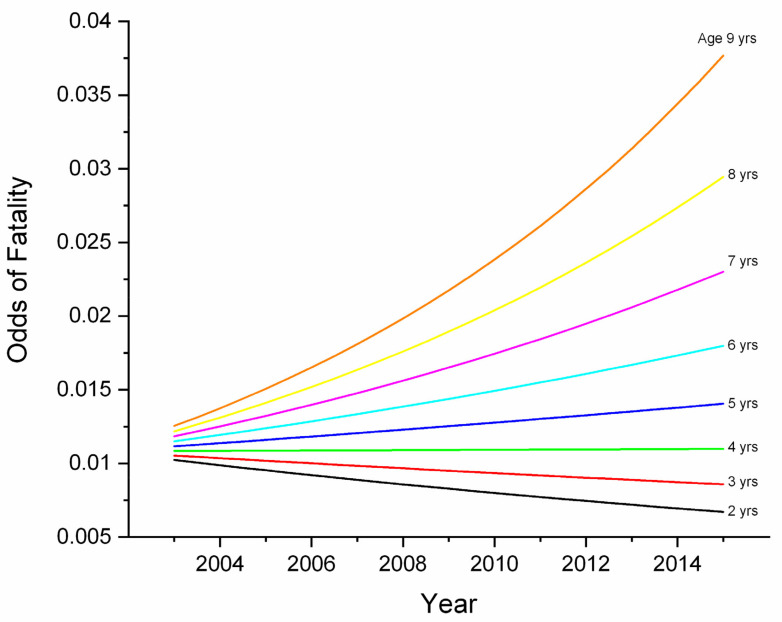
Odds of fatality by age and year for combined race-event and workout-event data for Ontario Thoroughbred racehorses for the period 2003–2015, unit of interest—work-event. This graph describes an AGE*YEAR interaction identified in multivariable logistic regression modelling of fatalities. For aged horses, odds increased over the study period, but for young horses, odds actually declined. This may be a reflection of increasing economic pressures throughout the study period.

AGE was significant as a main effect in race and workout models and was involved in interactions in workout and combined models. In the workout model, an increase in AGE of one year was associated with an increase in fatality odds of 1.4832 (1.0692–2.0576, *p* = 0.0182). In the race model, the effect was smaller (OR 1.1212, 1.0324–1.2176, *p* = 0.0066). In the combined model, a significant (*p* = 0.0213) but small interaction with year revealed odds to have decreased over the study period for young horses, but to have increased for older horses (Figure 1); a possible reflection of increasing economic constraints. An interaction between AGE and track/track surface (TS) in the workout model was only significant for comparison between turf at Track 1 and dirt surfaces at Track 1 (*p* = 0.0087) and Track 2 (*p* = 0.0369).

**Figure 2:**
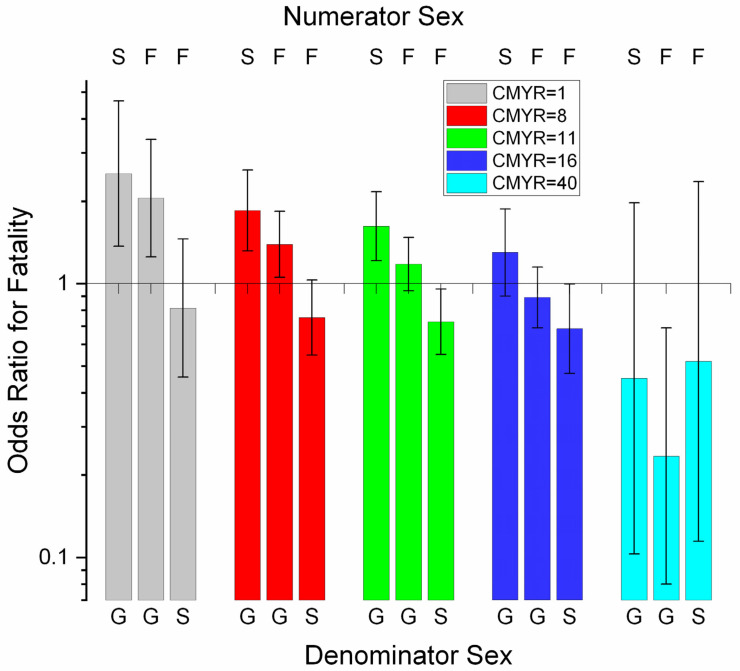
Odds ratios for fatality for a SEX*CMYR (sex by cumulative work-events for the season) interaction identified in multivariable logistic regression modelling of fatalities among Thoroughbred racehorses in Ontario for the period 2003–2015, unit of interest—race work-event. The graph describes pairwise comparisons between SEX groups according to values of CMYR. For OR values above 1, the numerator odds of fatality are higher than the denominator odds. Intact stallions consistently had the highest odds of fatality until high levels of CMYR were reached, when geldings showed the highest fatality. Females showed intermediate fatality odds. Confidence intervals that include 1.0 are non-significant. F—female; G—gelding; S—stallion. (Note log scale on *y*-axis.)

SEX was significant as a main effect in race and combined models but not for workouts. Fatality was less likely for geldings than females (race model, OR 0.4938, 0.2965–0.8223, *p* = 0.0067) and more likely for stallions than females (combined model, OR 1.3912, 1.1448–1.6907, *p* = 0.0009). However, there was a SEX*CMYR interaction in the race model (Figure 2). At low levels of CMYR, odds for geldings were consistently lower than for stallions or females, while odds for stallions were consistently higher than for females. At higher levels of CMYR, odds for geldings exceeded those for females, and at the highest levels also exceeded those for stallions. There was thus increasing liability with increasing work-event participation but with a sex differential, with liability transferring from intact horses to geldings at higher workloads.

**Figure 3:**
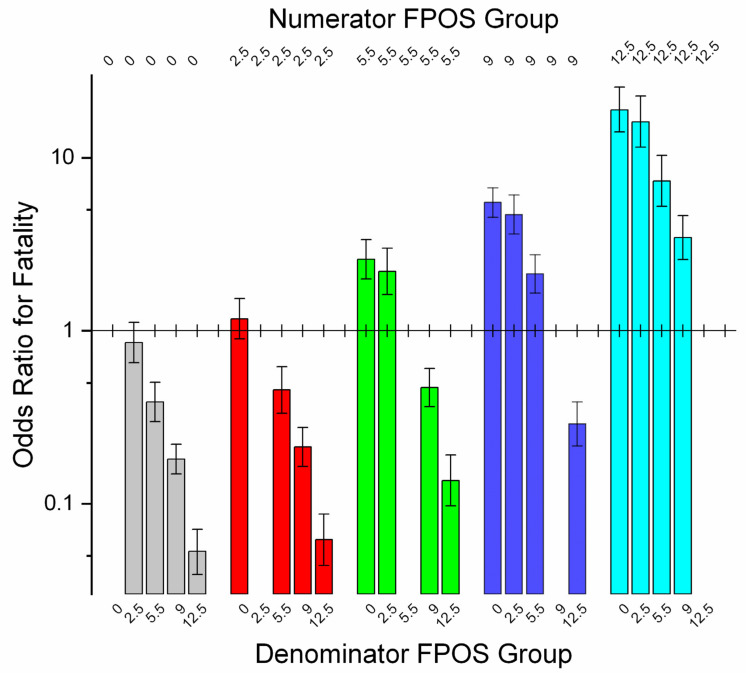
Contrasts between FPOS (finish position) groups for combined race- and workout-event data for Ontario Thoroughbred racehorses for 2003–2015, unit of interest—work-event, and showing odds ratios (OR) for fatality between groups together with their 95% confidence intervals. For OR values above 1, the numerator odds of fatality are higher than the denominator odds. Differences were greatest between FPOS 12.5 (race) and FPOS 0 (workouts, OR = 18.868, 14.085–25.641). Workouts and FPOS 2.5 did not differ. This pattern reflects, in part, increasing liability for late-finishing horses with increasing field size. Confidence intervals that include 1.0 are non-significant. (FPOS 0—workout event; 2.5—finish positions 1–4; 5.5—positions 5 and 6; 9—positions 7–11; 12.5—positions greater than 11). (Note log scale on *y*-axis.)

Finish position (FPOS) is only recorded for races. To bring workouts into a combined model and allow comparison of workouts with races, workouts were assigned FPOS = 0. FPOS was highly significant in race and combined models (Table 4 and Table S1, referent comparisons *p* < 0.0001 to *p* = 0.0133). As FPOS fell from FPOS 0 (workouts) to FPOS 12.5 (race finish position > 11, combined model), odds of fatality increased progressively. Contrasts revealed significant differences between all race FPOS categories and between workouts and all race categories (*p* = < 0.0001) except between workouts and FPOS 2.5 (Table S1, *p* = 0.2449), (Figure 3). Odds of fatality increased as finish position group fell.

**Figure 4:**
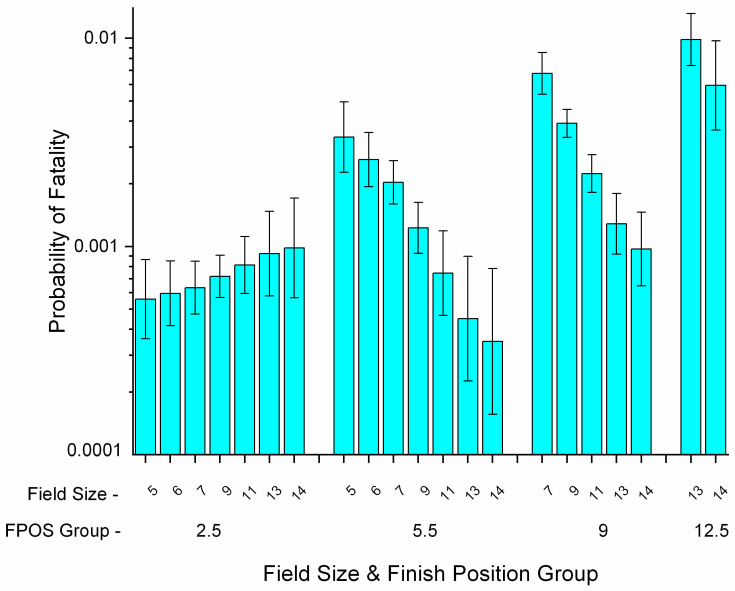
Probability of fatality for Ontario Thoroughbred racehorses for the period 2003–2015 according to results of multivariable logistic regression modelling, unit of interest—race work-event. The graph shows an interaction between finish position (FPOS) group and race field size (RSIZE). Probability of fatality showed a (non-significant) increase with RSIZE for horses in FPOS group 2.5 (positions 1–4). For all other FPOS groups, probability fell within group with increasing RSIZE leading to an overall protective main effect of RSIZE. (Note log scale on *y*-axis.)

Horses failing to finish a race, including fatal incidents, are placed in the last finish position, which depends on race field size (RSIZE) and ranged from 3 to 17. RSIZE was thus included in modelling. RSIZE was involved in an interaction with FPOS, with the slope of the interaction being significantly greater (increasing with RSIZE) for horses finishing in the first four positions (FPOS 2.5) when compared with the referent, 12.5 (decreasing with RSIZE, Figure 4). However, RSIZE was significant and protective as a main effect, with fatality falling by 0.5991 (0.4384–0.8186, *p* = 0.0013) for each increase in RSIZE of 1 (Table 4). Contrasts revealed the underlying effect to be complex.

Overall, probability of fatality rose with FPOS group (Figure 4), and for FPOS 2.5 there was a within-group (non-significant) increase in probability with increasing RSIZE. For all other FPOS groups, probability fell within FPOS group with increasing RSIZE (Figure 4). Thus, while liability increased as finish position fell, this increase was moderated within FPOS group by increasing field size, so that liability for midrange finish positions in larger races fell below that for FPOS 2.5 finishers. The latter showed an increase in liability, indicating that as field size increased, fatality liability started to transfer to early-finishing horses. After controlling for all other effects, the OR for a fatal outcome between a horse finishing FPOS 2.5 vs. 5.5 in a 5-horse race was 0.1660 (0.0932–0.2958), equivalent to that between a horse finishing FPOS 2.5 vs. 12.5 in a 14-horse race (OR 0.1647, 0.0794–0.3417). Overall, fatalities rose with field size and with low finish position. Race fatality rate by field size for non-last place finishers ranged from 0.3962/1000 (5 horse race) to 1.2019 (13 horse race). Rates for last place finishers ranged from 3.5528 (6 horse race) to 17.2684/1000 (12 horse race, Figure S1).

The dataset contained 27,985 separate races, with fatality taking place for horses placed in the last finish position in 233 or 0.8326% for an overall last place fatality rate of 8.326/1000 last place finishers. Mean fatality rate for all non-last finish positions was 0.9596/1000 non-last finishers, or 8.68 times lower. Of the 233 last place fatalities, 81.5% had an MSI and 12% died suddenly. Thus, of 433 race-event fatalities, 43.88% involved a horse placed last in its last race through injury that had a fatal outcome and 4.67% that were placed last died suddenly.

Fatality rates, distribution of presenting problem, and mean interval in days from last recorded work event to death are presented by FPOS group in Table 6. Fatality rates increased by group while mean interval and interquartile range (IQR) in days varied little. Presenting complaint was available for all but one case. Rates for MSI, dying suddenly, and colic were higher for horses in lower finish positions. MSI and dying suddenly combined were responsible for 93.22% of losses for FPOS 12.5. Of horses that died on the same day as their last recorded event (*n* = 236), 52 completed the event (7 coming in last) but 42 were found to have serious musculoskeletal problems post-race and were euthanised. Of these 42, 16 showed obvious signs before the end of the race, described as being eased (slowed down by the jockey) or fading. Ten (10) horses died suddenly immediately after the event. Of the 236 dying on their last race day, 175 were given last place finish positions but actually failed to finish their event.

**Figure 5:**
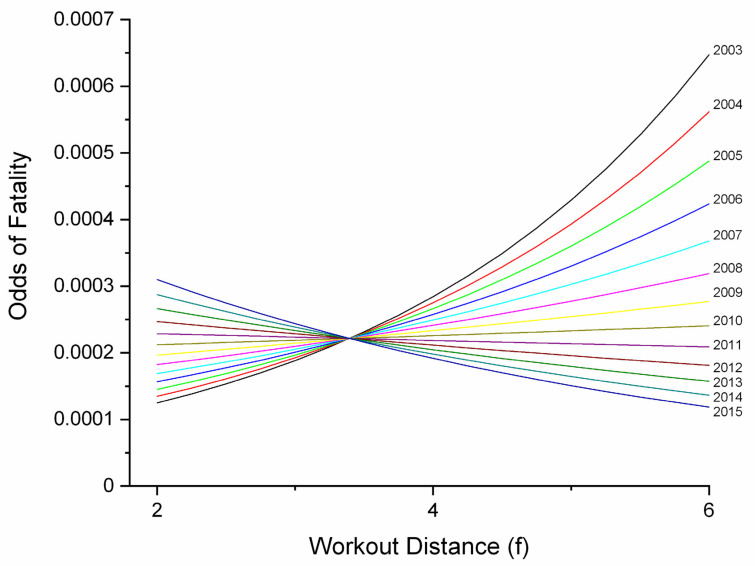
Odds of fatality by year (YEAR) and workout distance (DIST) for Ontario Thoroughbred racehorses for the period 2003–2015, unit of interest—workout work-event. The graph describes the interaction DIST*YEAR identified during multivariable logistic regression modelling of workout event fatalities. Fatality increased with workout distance early in the study period, but this effect disappeared by 2010, after which there was a reduction in fatality with increasing distance. Liability at 2f (mainly involving two-year-old horses) increased over the same period. (f—furlong.)

DIST (event distance) was not significant in the combined model but was involved in an interaction with DOWK in the race model (see below). DIST was significant as a main effect in the workout model, with each increase of 1 furlong in workout distance being associated with an increase in odds of fatality of 1.5091 (1.2034–1.8925, *p* = 0.0004), while DIST and YEAR were involved in an interaction, with a rise in fatality as workout distance increased early in the study period falling with successive years until the effect reversed by 2010 (*p* = 0.0027, Figure 5). Over the same period, fatality in the shortest workouts increased. Since the shortest workout distances tend to be for younger horses early in the season (mean AGE for 2f workouts 2.79 ± 1.17 years vs. 3.46 ± 1.36 for distances > 2f, mean ± std. dev.), this finding is another dimension of the liabilities facing young horses. There was no change in mean or range for workout distance over the study period. A total of 2.04% of workouts were held at distances greater than 5.5 furlongs and were associated with 2.78% of workout fatalities (rate 0.7478/1000 workouts), while workouts at 5.5 furlongs or less (97.96%) were associated with 97.22% of workout fatalities (0.5449/1000).

**Figure 6:**
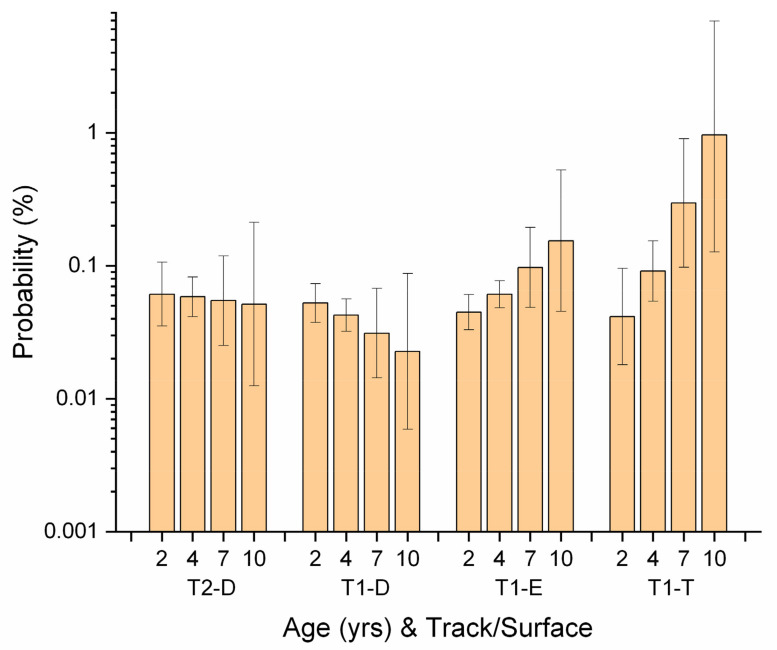
Probability (%) of fatality for workout work-events for Ontario Thoroughbred racehorses for the period 2003–2015, unit of interest—workout work-event, and describing an interaction between horse age (AGE) and track/surface combination (TS, AGE*TS) identified during multivariable logistic regression modelling. Track/surface combinations were used because not all surfaces were available at both tracks. On dirt surfaces at both tracks there was a non-significant trend to decreasing probability as AGE increased, but for Track 1 synthetic and turf surfaces, probability of fatality increased with AGE. (Note log scale on *y*-axis.) T2-D—Track 2 dirt; T1-D—Track 1 dirt; T1-E—Track 1 synthetic; T1-T—Track 1 turf.

There was no effect of TRACK on fatality in the race or combined models. Because of differences in available surfaces, with workouts only taking place on one surface at Track 2, TRACK and SURF were combined into a single variable, TS. There was no main effect for TS but there was a significant interaction with AGE in the workout model for which Track 1 turf workouts had significantly higher fatality than dirt workouts (Track 1 dirt OR 0.6072, 0.4182–0.8815, *p* = 0.0087; Track 2 dirt OR 0.6599, 0.4467–0.9750, *p* = 0.0369). A pattern of increasing probability of fatality with increasing AGE for turf and synthetic workouts is illustrated in Figure 6. This interaction was the only appearance of track surface in any model and may reflect quality of entries and intensity of competition for different track/surface combinations rather than any inherent characteristic of surface. Raw race-event fatality rates by track/surface combinations and distance are shown in Figure S2. Distance only appeared in a DIST*DOWK interaction for Sunday vs. the referent Wednesday in the race-events model.

Day of week (DOWK) appeared as a main effect in the combined model, with fatality being significantly more likely to take place on a Thursday than a Wednesday—the referent (*p* = 0.0066). In the race model there was a DIST*DOWK interaction. Odds ratios revealed fatality in longer races to be higher on Sunday (both tracks) and Tuesday (Track 2) than on all other days, while fatality in short races was relatively low on those days. For Sunday, each 1 furlong increase in race distance was associated with a fatality OR of 1.3636 (1.0174–1.8275, *p* = 0.038). This reflected unusually high fatality in a small number of races. Fatality rate was highest in races > 10 furlongs with fatality range for these races being 2.1322–10.5264 fatalities/1000 races > 10f, but DIST was not significant as a main effect in modelling of race events. DOWK was not significant in the workout model. 

Feature and high purse races are more likely to be held on a Sunday and to be longer, involving more successful and potentially older horses. Tuesday racing took place almost exclusively at Track 2. Races at 2, 9.5, and 11 furlongs at that track had relatively high fatality (5.22, 7.46, and 14.29 deaths/1000 starts, respectively (Figure S2), vs. a track race average of 2.02). Fatality was high at Track 1 at 12, 13, and 14 furlongs (3.2, 13.33, and 15.87 deaths/1000 starts, respectively, vs. a track race average of 1.75). Fatality tended to be high in the shortest races on Monday (both tracks), and Wednesday and Thursday (Track 1). The statistically significant higher overall fatality on Thursdays in the combined model reflected primarily fatalities in short races at Track 1. Quarter mile sprints were discontinued at Track 1 after 2010. DOWK effects reflect track characteristics such as scheduling of racing and variations in the quality of competition, public attendance, and racing in other jurisdictions that simulcast their product. Race year (YEAR) was significant in the workout model, with fatality increasing by year (OR 1.2025, 1.0363–1.3953, *p* = 0.0151).

CMD (cumulative days raced) did not appear in the workout model but increasing CMD was associated with increasing fatality in both race (OR 1.0491, 1.0175–1.0816, *p* = 0.0021) and combined (OR 1.0245, 1.0105–1.0388, *p* = 0.0006) models. Interaction between CMD and TRACK in the combined model, with fatality being more likely to occur with higher CMD at Track 2 than Track 1, may reflect difference in AGE distribution, with Track 2 having more older horses (mean age 4.34 ± 1.58 years vs. 3.41 ± 1.29, mean ± std. dev. for Track 1) and a higher 50th percentile for CMD (76 vs. 67), though the CMD*TRACK effect was small (OR 1.0308, 1.0033–1.0591, *p* = 0.0278). Track 2 racing is less intense and generally involves less successful horses than Track 1.

Cumulative events for the year (CMYR) entered the race work-event model as an interaction with SEX for geldings vs. females (Figure 2), suggesting cumulative time in training (CMD) may be more impactful than number of events. Cumulative career events (CMCAR) was significant in all three models and was associated with decreasing odds of fatality. This effect could indicate both a progressive positive impact of training and experience and a survival effect, whereby less robust horses have been withdrawn from competition.

### 3.2. Modelling by Horse-Year

Results of modelling fatality associations by horse-year are presented in Table S2 (race work-events) and Table S3 (workout work-events). Analysis addressed the last workout and race work-event for each horse in each year the horse worked. Raw fatality rates were 9.7 and 4.77/1000 horse-years for races and workouts, respectively. Overall fatality (race and workout fatality combined) expressed with race horse-years as the denominator was 15.3453 deaths/1000 race horse-years.

**Figure 7:**
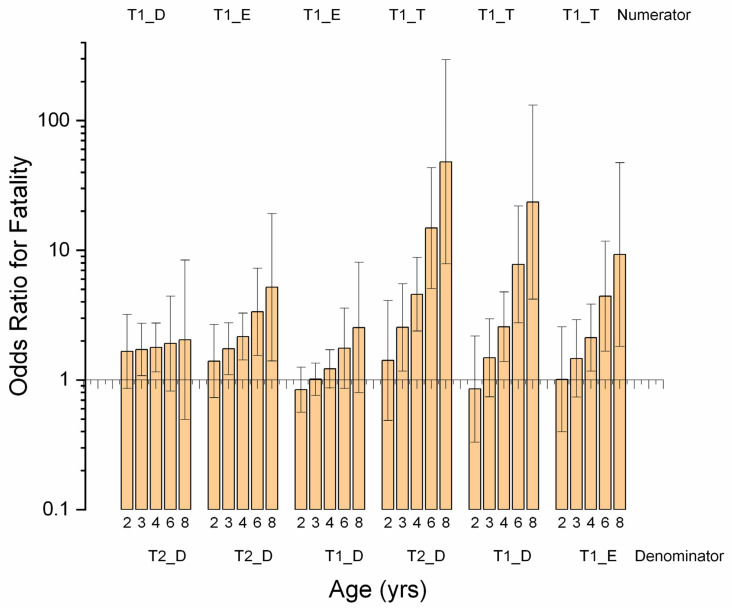
Odds ratios (OR) for contrasts between combinations of track and surface (TS) and horse age (AGE) and describing an AGE*TS interaction identified in multivariable logistic regression modelling of fatalities in Ontario Thoroughbred racehorses for the period 2003–2015, unit of interest—workout event by horse-year. For OR values above 1, the numerator odds of fatality are higher than the denominator odds. The pattern for each comparison is for OR to rise as AGE increases and reflects progressively increasing fatality with age in the numerator. The effect is most marked for workouts on turf at Track 1, which show a very high liability for older horses. No workouts were recorded on turf at Track 2. (Note log scale on *y*-axis.) Confidence intervals that include 1.0 are non-significant.

AGE was not significant in either horse-year model, but there was an AGE*TS interaction in the workout model (Figure S3). Probability of workout fatality decreased with AGE on all combinations of track/surface when compared to turf surface at Track 1. Contrasts revealed odds ratios for workout fatality on turf to be higher than on dirt or synthetic and for the difference to increase with age (Figure 7). A similar interaction was found in the workout work-event model (Figure 6). Surface was not significant as a main effect in any model in this study, and neither AGE nor SURF were significant in the horse-year race model (Table S2). Since turf racing tends to involve a higher quality of horse on Ontario tracks, these findings suggest qualifying for a change in race surface to turf may be accompanied by unusual risk for older horses.

SEX was only significant as a main effect in the workout model and only for stallions compared with the referent—females (OR 1.5625, 1.1691–2.0884, *p* = 0.0026). FPOS was highly significant in the horse-year race model for all referent comparisons except FPOS 9 (finish positions 7–11, *p* = 0.1012), with odds of fatality increasing progressively as FPOS fell. SEX interacted with FPOS in the race model, but the interaction was only significant for comparison between geldings finishing in FPOS 2.5 (low fatality) and females in FPOS 12.5 (OR 3.0581, 1.4092–6.6365, *p* = 0.0047). 

Race year (YEAR) was significant as a main effect in the horse-year race model, with fatality decreasing each year (OR 0.9696, 0.9437–0.9963, *p* = 0.0259). There was an effect of TRACK in the horse-year race model (*p* = 0.0032) describing lower fatality at Track 2 than Track 1. Though this may have reflected a horse effect, there being movement between tracks, this could not be pursued in the present analysis. Day of week (DOWK) was not significant in the horse-year analyses. 

**Figure 8:**
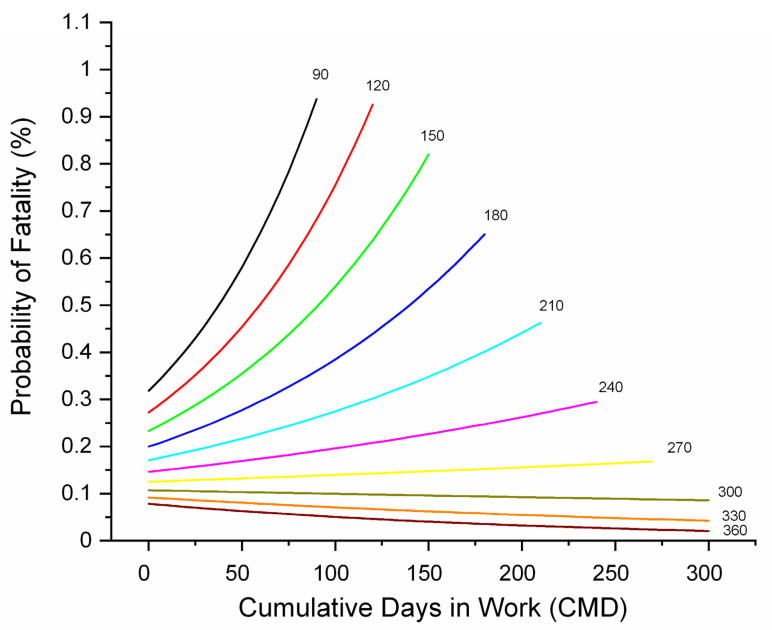
Probability of fatality (%) by cumulative days in work (CMD) and yearday (YD) and describing a CMD*YD interaction identified during multivariable logistic regression modelling of associations with fatality for Ontario Thoroughbred racehorses for the period 2003–2015, unit of interest—race-event by horse-year. YD for each relationship is shown at the end of each curve. At the start of the season, probability is higher and there is a relatively rapid increase in fatality with increasing days in work, but as the season progresses, liability falls and the gradient of this relationship progressively decreases, then reverses. There was no second order relationship with CMD.

In the horse-year race model, CMD was highly significant, with increasing CMD being associated with increasing fatality (OR 1.1922, 1.0964–1.2963, *p* < 0.0001), and it was also involved in an interaction with YD (Figure 8). For horses with limited days in work early in a season, fatalities accumulated relatively rapidly and were high. As the season advanced, the fatality rate fell progressively. High liability early in the season may reflect stage of training and lack of familiarity with the track environment. Cumulative events for the year (CMYR) only entered the workout model, with an increasing number of workouts being protective. The effect of RSIZE in the horse-year race model closely mirrored that in the work-event race model.

RWYN was significantly associated with increasing fatalities in both the race and workout horse-year models, indicating that fatalities occurred in horses with higher numbers of races or workouts. There was a second order effect in the race model, however, the rate of accumulation of fatalities increasing as RWYN increased until reaching a peak at RWYN = 8.15, followed by decreasing fatalities as RWYN increased beyond 8.15. Since RWYN contained a number of race starts in the race model, this finding indicates that on average, horses that started more often than this were subject to a decreasing fatality liability with increasing work. This effect parallels the CMD*YD interaction described above. Horses undertaking an increasing number of workouts had a progressively increasing fatality liability, however, suggesting the possibility of ongoing problems.

## 4. Discussion

This study focuses on general fatality within a 60-day window, though MSI predominated in presenting problems with the interval between work and death being short and with a high exercise association and MSI-related fatality rate [27]. There is little information in the literature on general fatality in racehorses, while it is not always clear from published reports whether case definitions encompass instances other than on-track, race-day deaths, and MSI requiring euthanasia. Equally, the window between exercise and death within which a case is captured varies from study to study. Comparison between the present report and other studies should thus be with caution.

Rates for catastrophic musculoskeletal injuries among Thoroughbred racehorses across racing jurisdictions globally were recently summarised [7], with effect sizes ranging from 0.29 [30] to 2.36 [63]/1000 race starts. Comparison between studies is complicated by differences in jurisdiction, reference population, case definition, sampling frame, and denominator used in calculating rate and study size [58], but rates for Ontario Thoroughbred racing expressed on the same basis as above are high (MSI only) or very high (all fatalities). Rates based on MSI and actual race starts currently provide the most reliable basis for comparison, though from a societal perspective, rates reflecting overall fatality may provide the more meaningful basis for response. Rates based on stratified groups (for example, by race or horse characteristic) together with associations derived from modelling provide the best guidance for application of focused strategies. Though modelling is aimed at causal inference, it may be unrealistic to expect the methodology will ever reveal more than contributing factors in what is essentially a complex system.

While descriptive analysis identified age and sex as major contributing factors to fatality in this dataset [27], multivariable analysis revealed subtleties; these have been previously observed (see Hitchens et al., 2019) [7]. The associations are complex. Young intact male horses have a high fatality rate, but the underlying associations appear to include experience and stage of preparation, with inexperience and limited time in training both contributing to high early fatality. Results indicate fatalities accumulate more rapidly early in the season in young horses than later. Geldings appeared better able to benefit from accumulating work-events than stallions or mares, even though they had more juvenile starts and thus event risk. This suggests behaviour/temperament may be a more significant factor than training miles for intact animals early in the season. Intact animals may also be managed differently in relation to training distance and time, leading to a differential in physical preparation, with the impacts being masked by sex-linked differences in an animal’s approach to work. Geldings appear to carry the major risk as they age and are more likely to continue in work than intact animals.

Finish position and field size were both associated with fatality, but with opposite effects. Mechanisms for an impact of finish position include the practice of awarding last place positions to horses failing to finish and the influence on performance of ongoing health issues, with some having the potential to contribute to fatality. Reasons for an opposing influence of field size and the pattern of interaction with finish position are less clear. An impact of field size on injuries has previously been observed, though in those analyses it was for injuries to increase with field size [30,43,44,64] and was possibly complicated by associations between field size and other race characteristics such as distance and surface [19]. A negative association was revealed here when both finish position and field size were included in the model, partially controlling for the practice of awarding last place positions to horses not finishing, many of which may be fatalities. Fatality liability does increase with field size, but the effect is not uniform. Findings suggest being in the middle of a large field may be relatively safe. The effect of field size on liability for early-finishing horses is interesting and may reveal an impact of the effort involved in getting to the front and staying there. Conversely, for following horses, a larger field may provide more opportunity to settle into the pace.

There are also likely to be other influences not measured in this study. It has been suggested that the effect of field size may reflect the observation that field sizes tend to be greater in turf races and that these tend to have higher fatality [7,19]. Surface had limited impact on race fatality in the present study, but interactions were found with distance and age. Descriptive analysis of raw data revealed a modest peak of fatality in races below 5 furlongs and with medium to small field sizes, but that the greatest fatality was associated with races 9–12 furlongs and with intermediate field size (8–11 horses). These findings suggest strategies employed during a race might influence probability of fatality, and in either direction. Opportunities for a jockey to make adjustments on the basis of circumstances at the start of a race, including how well a horse left the gate and post position [30], will be influenced by both race distance and field size as well as horse characteristics. These factors have been observed to influence race-day jockey falls [65], and may also influence race-time decision-making. There may be less pressure on jockeys to make decisions in races with larger fields. These findings are worthy of further examination.

In an analysis of standardbred fatalities, horses that did not finish (DNF) could easily be differentiated from those finishing late, since race records identify DNF in the race line and a finish position is not assigned. Identification of an association between finish position and liability to fatality in non-DNF horses in that study was thus independent of the influence of horses that failed to finish [66]. For the race events in the present analysis, reference must be made to full race charts to identify reasons for a horse coming last, while in some races there may be more than one DNF horse and each would have been given a sequential numeric finish position. Available resources did not allow evaluation of all race charts in the present study, and the fatality association with finish position identified here is almost certainly heavily influenced by DNF horses. It cannot be stated with confidence, therefore, that there is an association between finish position and probability of fatality that is independent of horses that failed to finish.

Indices of workload and intensity used in this study addressed overall horse exposure and not strict relationship to fatality. Associations found both agree and contrast with previous investigations of MSI. Increasing cumulative career work-events (an approximation to career length) was protective against fatality in all models. Number of seasons racing was also observed to be protective for New York horses [28]. The source of these associations cannot clearly be apportioned between a positive impact of training, competition, and experience on robustness, a survival effect whereby less capable horses have been withdrawn, or a combination thereof. Likely both influences are operating. Other studies found equivocal or no relationship of career length with injury [39,44,67].

Other indices of career length/cumulative work have been applied in studies focusing on specific MSI with generally positive associations [21,56,68]. Acknowledging the tendency to higher fatality in younger inexperienced horses, the association identified here might be expected to produce a higher order relationship [44,58], but no significant second order effects were identified for work indices except for RWYN in the horse-year race model. Here, fatality initially increased with increasing races but then levelled off and fell at the highest levels of RWYN, consistent with benefits accruing from accumulating training and experience. 

Weak associations with CMYR, cumulative work-events for the season, may have resulted from use of a statistic that combined race and workout events. In the horse-year models, the variable RWYN was specific to either workouts or races and had more clear model impacts. This and the observed differences in fatality rates suggest work indices for workouts and races should to be treated entirely separately, since they may have conflicting effects that may obscure associations. Equivalency between races and workouts/training when assessing workload ought not to be assumed. 

Cumulative days in work (CMD) only appeared in the race models and its impact was limited. The variable may more effectively encompass total time in work by season, including training, rather than work-events only, but may be insensitive because it does not account for temporary absences/layoffs, which have been found to be impactful in some studies [19,69]. It also cannot discriminate between type or quantity of work. Despite this, increasing fatality with increasing time in work does parallel the effect of RWYN in the horse-year models, and similar effects have been observed in other studies [25,26,46]. This could be a direct result of work-related wear and tear and total days at risk, but the second order effect for race starts in the horse-year model indicates that for horses racing frequently and successfully in the present study, fatality liability fell at higher workloads, possibly both a training and survival effect. The effect may also reflect more critical selection of those horses able to thrive in the racing environment and compete successfully, as opposed to their being eliminated earlier in their careers by injury or limited performance. This study finding supports the value of analysis at horse- rather than only event-level.

Because of differences in workout and race models, primary emphasis was placed on these analyses and not on the combined model. However, observations were made in the latter that provide interesting insights. Increasing liability for older compared with younger horses may have reflected increasing industry economic constraints during the study period. These were associated with recession-related influences and changes in provincial financial support. There was a significant contraction in the industry with a one-third reduction in the number of horses competing after 2010. This contraction raises potential welfare issues since horses may have been removed from racing that would otherwise have been retained, while elective euthanasia may have been selected more readily rather than attempt treatment in cases of injury in older horses. A gradual reduction in fatalities was evident over the study period among horses actively engaged in racing, but this would not account for losses among horses withdrawn from racing. 

Historically, race days and feature races have changed over time at both tracks, and no consistent patterns involving DOWK could be identified beyond the differential between Monday and Thursday. Though workouts took place at both tracks on a Thursday, racing only took place at Track 1. Reasons for the effect are unclear but may involve a lower calibre of racing and more horses with problems. This finding may therefore provide some opportunity for strategic intervention. Association with cumulative days worked for Track 2 may reflect a higher number of horses with problems attempting to qualify, though the effect size was small and wide variation in numbers from year to year could make this a limited target for intervention. Additional track and management factors that might help explain findings and be worthy of study include track quality, race number, and number of races per race day and time of day at which a meet is held.

Statistics were generated here with the horse as the unit of interest. Fatality represents a production and operational cost that, to the economic welfare of the industry, is as important as are welfare concerns to the general public. The horse and all of the mechanisms involved in facilitating and supporting its active participation in competition are of paramount importance and represent the true complexity of the industry. Though representing the “sharp end” of the activity of racing and its ultimate expression, races only punctuate a very extensive venture. Singular focus on the race as the unit of interest might do a disservice to the broad sweep of racehorse management and preparation. From a management as well as a welfare perspective, therefore, morbidity and layoff data would complement fatality data in achieving optimal resource management.

There are several instances in analysis of this data set in which variables that did not prove significant in modelling showed contrasting effects in descriptive analysis. This illustrates the importance of controlling for all possible sources of influence when identifying associations but does not invalidate descriptive results since these can point to clusters where strategic interventions could be of benefit. This might also be an argument in favour of caution when using univariable analysis to determine which explanatory variables to include in multivariable modelling [61]. Examples of such instances are the unusually high fatality associated with 2 furlong and distance races, even though distance was not significantly associated with race fatality, and the conflicting and complex relationship between race field size and fatality. The high fatality rate for young stallions is another example. Many factors may have contributed to the outcome in these situations, causing distance not to appear significant, field size to have contradictory associations, and de-emphasising high liability for young, intact horses. These factors include missing data. Group sizes and limited opportunity to stratify data, plus concerns regarding over-fitting will often preclude dealing with such clusters during modelling. Focusing on the circumstances of the horses involved in such clusters is likely to have a high yield in reducing fatalities.

In the context of MSI, much has been made of the significance of track surface [20,25,26,28,36,45,55,70,71,72,73,74,75]. This was especially a focus of interest at Track 1 with the elimination of dirt surface racing and the introduction of a synthetic track surface. The present analysis only identified a clear effect of track surface when analysing workouts, with turf carrying a higher risk, and these may have been horses moving from dirt or synthetic to turf racing. Relatively few workouts are performed on turf at the subject tracks. The present study addressed general fatality, and different results might have been obtained with primary focus on MSI. With this proviso, there was no clear evidence in this study of an effect of surface on fatality in races.

Findings point to impacts of youth, sex, inexperience, and early training for young horses and to cumulative effects for older horses, with geldings carrying much of the liability as they age. Results also suggest that fatalities might be reduced by targeting high-risk groups, enhanced pre-race inspection (especially for horses that have been finishing poorly), and increasing understanding of the way in which jockey/race strategy might impact outcome. Advantages could also accrue from gathering information on horses that are withdrawn from racing, even if those withdrawals are not clearly linked to specific clinical issues. The high incidence of horses dying suddenly represents a particular issue and a problem whose cause requires focused investigation. The issues for these cases are likely to be highly dynamic and to have substrate and trigger dimensions, both of which could be further investigated by appropriate monitoring strategies. The potential for human injury also cannot be ignored.

It is of interest that in the present dataset neither surface nor distance appeared in the race model as main effects, suggesting that some other variable may be of importance in studies in which these factors were found to be significant. It seems logical that these variables may simply represent an opportunity for a more common, underlying influence to be expressed. This may be the stress of racing and race preparation. In this interpretation, the independent variables that represent the greatest sources of stress in any particular dataset will be the ones that show the greatest association with adverse outcomes. This represents a different model or approach when attempting to understand the causes of adverse outcomes.

### Limitations

Of 695 fatalities, 10 could not be analysed on the basis of officially recorded work. It was not possible to state with clarity what portion of fatalities may have been associated with training rather than work-events. Achieving clarity here represents a direction for expansion of the adverse events program. The 60-day horizon for collection of fatality data mandated by the province’s Rules of Racing does not capture all fatalities, especially those taking place off-track in horses in early training. This number may be small for the Thoroughbred but does represent a limitation in assessing overall losses among horses in training. No information was available on morbidity. Resource limitations precluded full analysis of race charts. Additional relevant information may be available from that source. Despite close temporal relationships between fatality and officially recorded work-events, some fatalities may not have been influenced by recent work. At the upper limits of most variables’ ranges, group sizes became small and estimates became less reliable. This needs to be considered when interpreting findings. Although indicative of fatality-associated wastage in a provincial Thoroughbred racing community, the present results should be applied with caution to other racing populations.

## 5. Conclusions

Comparison between present data and published literature indicates fatality rates for the Ontario Thoroughbred to be high. There is no single explanation. Here, as in previous studies, musculoskeletal injury was the predominant complaint, but horses dying suddenly was also a frequent complaint. Liability is high for young horses early in the season with a differential that varies by sex. These findings present opportunities for intervention. Higher liability for horses switching from dirt/synthetic to turf racing and for young horses engaged in short-distance sprints present additional targets. Though the impact of race distance appears limited, high mortality in some large field races, when combined with effects of age and workload, identify circumstances where horses might be at particular risk. Large races run at distance tend to be feature races, and fatalities particularly noticeable. Relationships between field size, finish position, and fatality are complex, but provide additional insight into possible causes of loss and focus for intervention; the greatest proportional impact in large races may be on the race leaders. 

Horses undertaking repeated workouts appear to have a higher liability, suggesting ongoing problems and a need for closer monitoring. Evidence jockey strategy could be a contributing factor in fatality reveals need for further study. This investigation could not confirm a specific impact of training, but results suggest rapid accumulation of workload in animals in early preparation is likely damaging, and that the role played by workload and environment in general as sources of physical and psychological stress requires examination. Though fatality appears to fall toward the end of a season and for horses with a long career history of successful performance, it is horses that do not exhibit this robustness and staying power that represent the population of greatest concern in reducing fatality. If identified associations represent sources of stress, current or cumulative, then identifying at-risk animals on this basis may be as productive as targeting specific, discrete mechanisms suspected to be causative.

It will be up to the industry and regulators to decide what aspects, if any, of this study’s findings they wish to incorporate. Handling and management of young horses and impacts of sex may require fundamental changes in the industry at numerous levels. Close monitoring of musculoskeletal function, evaluating horses in greater depth before they work or race, and ensuring injured horses are recovered before return to work, applied as extensions to the close monitoring many trainers already apply, might be more rapidly adopted, but have manpower and resource dimensions. Achieving a better understanding of reasons for horses dying suddenly, though urgent, likely has a long horizon. Specifics aside, many industry members are already concerned by injury and fatality levels and welcome industry-wide initiatives acknowledging and addressing the problem. Clearly, we need to know more.

## Figures and Tables

**Table 1:** Definition of abbreviations and variables used in analysis of fatality data for Ontario Thoroughbred horses, 2003–2015.

Variable	Definition	Term or Range	Type	Notes
AGE	age	2–14 years	continuous	
SEX	sex for work-event	F-female/G-gelding/S-stallion	categorical	
DOWK	day of week for work-event	Friday-Thursday	categorical	
DATE	date of work-event	18 February 2003–29 November 2015	date	
RSIZE	race field size	3–17	continuous	
SURF	track surface	dirt (‘D’), synthetic (‘E’), turf (‘T’)	categorical	
TRACK	racetrack for work-event	Track 1 (T1), Track 2 (T2)	categorical	
TS	track-surface combination	T1-D, T2-D, T1-E, T1-T, T2-T	categorical	
DR	fatality	0—NO, 1—YES	binary	model outcome
DIST	race or workout distance	furlongs, f	continuous	
FPOS	race event finish position group	2.5 (1–4), 5.5 (5, 6), 9 (7–11), 12.5 (>11)	categorical	
RNUM	race number for date	1–15	continuous	
TYPE	work-event type—race or workout	race (R), workout (W)	categorical	
YD	yearday	1–365 (/30 in models)	continuous	work-event day of year
YEAR	calendar year	0–12 (2003–2015)	continuous	
YOB	year of birth	1991–2013	continuous	
CMCAR	cumulative career races + workouts to current work-event	0–202 (/10 in models)	continuous	career
CMYR	total races + workouts in season to current work-event	0–40 (/10 in models)	continuous	season
CMD	cumulative days in work from first to current work-event	0–294 (/10 in models)	continuous	season
RWYN	total races OR workouts	1–21 (race), 1–32 (workouts)	continuous	season, horse-year models
AGCO	Alcohol and Gaming Commission of Ontario			
CI	confidence Interval			
OR	odds ratio			

**Table 2:** Distribution of presenting complaints and results of consolidation for Ontario Thoroughbred horse fatalities, 2003–2015.

PROBLEM		Count	Total	%
Presenting	Consolidated			
Fracture		338		
Catastrophic soft tissue injury		123		
Exertional rhabdomyolysis		2		
	MSI	463	463	66.62
Collapse		9		
Dropped Dead		63		
Coronary Artery rupture		1		
Heart attack		6		
Found dead		10		
	Died Suddenly	89	89	12.81
Colic		41		
	Colic	41	41	5.90
Medical complaint		8		
Laminitis		2		
Diarrhea		4		
Respiratory problem		6		
Renal disease		1		
Bacterial infection		3		
	Medical	24	24	3.45
Septic arthritis		2		
Medication reaction		13		
	Iatrogenic	15	15	2.16
Self-inflicted trauma		10		
Off-Track accident		16		
On-track accident		13		
	Accidents	39	39	5.61
Neurological		17		
	Neurological	17	17	2.45
Epistaxis		4		
Severe hemorrhage		2		
	Haemorrhage	6	6	0.86
Unknown		1		
	Unknown	1	1	0.14
Total			695	100

Presenting—entered on Submission Sheet. Consolidated—presenting complaints consolidated to 9 groups. MSI—musculoskeletal injury.

**Table 3:** Fatality rates in Ontario thoroughbred racing 2003–2015 according to different methods of calculation and by consolidated presenting problem *.

Numerator	All Fatalities	All Fatalities	Workout Fatalities	Race Fatalities
Denominator	Race events	All Events	Workout events	Race events
Overall	2.9401	0.9994	0.5708	1.8317
MSI	1.9587	0.6658	0.3529	1.2733
Died suddenly	0.3765	0.1280	0.0784	0.2242
Colic	0.1734	0.0590	0.0436	0.0888
Medical	0.1015	0.0345	0.0196	0.0635
Iatrogenic	0.0635	0.0216	0.0131	0.0381
Accident	0.1650	0.0561	0.0436	0.0804
Neurological	0.0719	0.0244	0.0152	0.0423
Hemorrhage	0.0254	0.0086	0.0044	0.0169
Unknown	0.0042	0.0014	0	0.0042

* Rate per 1000 denominator events. MSI—musculoskeletal injury.

**Table 4:** Logistic Regression Modelling of Associations with Equine Fatality in Ontario Racing for Thoroughbred Race Events, by Event, 2003–2015.

Events	236,386					
Fatalities	433				Conf. Int.	
Variable	Estimate	s.e.m.	*p*-value	OR ^†^	Lower	Upper
Intercept	3.0303	2.2249				
AGE	0.1144	0.0421	0.0066	1.1212	1.0324	1.2176
SEX, G vs. **F**	−0.7057	0.2602	0.0067	0.4938	0.2965	0.8223
SEX, S vs. **F**	0.2087	0.3108	0.5019	1.2321	0.6700	2.2657
SEX*CMYR, G vs. **F** ^†^	0.5392	0.1881	0.0041	1.7146	1.1859	2.4791
SEX*CMYR, S vs. **F** ^†^	0.1353	0.2524	0.5920	1.1449	0.6981	1.8776
FPOS, 2.5 vs. **12.5**	−9.8570	2.0706	<.0001	0.0001	0.000001	0.0030
FPOS, 5.5 vs. **12.5**	−6.4891	2.0789	0.0018	0.0015	0.000026	0.0894
FPOS, 9 vs. **12.5**	−5.0902	2.0561	0.0133	0.0062	0.0001	0.3464
RSIZE	−0.5124	0.1593	0.0013	0.5991	0.4384	0.8186
RSIZEˆFPOS, 2.5 vs. **12.5**	0.5753	0.1665	0.0005	1.7777	1.2827	2.4636
RSIZEˆFPOS, 5.5 vs. **12.5**	0.2608	0.1707	0.1265	1.2980	0.9289	1.8137
RSIZEˆFPOS, 9 vs. **12.5**	0.234	0.164	0.1537	1.2636	0.9163	1.7427
DIST (f - furlongs)	−0.2019	0.1403	0.1501	0.8172	0.6207	1.0758
DOWK, Fri vs. **Wed**	−1.7054	1.2647	0.1775	0.1817	0.0152	2.1671
DOWK, Sat vs. **Wed**	−0.8539	1.1024	0.4386	0.4258	0.0491	3.6943
DOWK, Sun vs. **Wed**	−1.9014	1.0293	0.0647	0.1494	0.0199	1.1230
DOWK, Mon vs. **Wed**	−0.1059	1.1431	0.9262	0.8995	0.0957	8.4535
DOWK, Tue vs. **Wed**	−1.8266	1.1241	0.1042	0.1610	0.0178	1.4574
DOWK, Thu vs. **Wed**	0.8666	1.3217	0.5121	2.3788	0.1784	31.7259
DIST*DOWK, Fri vs. **Wed** ^†^	0.2169	0.1825	0.2347	1.2422	0.8687	1.7764
DIST*DOWK, Sat vs. **Wed** ^†^	0.1418	0.1610	0.3784	1.1523	0.8405	1.5799
DIST*DOWK, Sun vs. **Wed** ^†^	0.3101	0.1494	0.0380	1.3636	1.0174	1.8275
DIST*DOWK, Mon vs. **Wed** ^†^	0.0527	0.1699	0.7566	1.0541	0.7556	1.4706
DIST*DOWK, Tue vs. **Wed** ^†^	0.2968	0.1619	0.0668	1.3455	0.9797	1.8480
DIST*DOWK, Thu vs. **Wed** ^†^	−0.0620	0.1950	0.7504	0.9399	0.6413	1.3774
CMD (/10)	0.0479	0.0156	0.0021	1.0491	1.0175	1.0816
CMYR (/10)	−0.3568	0.2224	0.1086	0.6999	0.4526	1.0823
CMCAR (/10)	−0.1341	0.0307	<0.0001	0.8745	0.8234	0.9287

^†^ Result is a ratio of odds ratios. AGE—age, years; SEX—sex, F-female, G-gelding, S-stallion; DOWK—day of week for work-event; RSIZE—race field size; DIST—race or workout distance, furlongs, f; FPOS—race event finish position group, 2.5 (1–4), 5.5 (5, 6), 9 (7–11), 12.5 (>11); CMCAR—cumulative career races + workouts (divided by 10); CMYR—total races + workouts in season to current work-event (divided by 10); CMD—cumulative days in work from first to current work-event (divided by 10). Referents for categorical variables are in bold.

**Table 5:** Logistic Regression Modelling of Associations with Equine Fatality in Ontario Racing for Thoroughbred Workout Events, by Event, 2003–2015.

Starts	459,013					
Fatalities	252				Conf. Int.	
Parameter	Estimate	Error	*p*-value	OR	Lower	Upper
Intercept	−9.8117	0.8831				
AGE	0.3942	0.1670	0.0182	1.4832	1.0692	2.0576
TS - T2_D vs. **T1_T**	1.2173	0.8511	0.1527	3.3781	0.6371	17.9117
TS - T1_D vs. **T1_T**	1.2314	0.7810	0.1149	3.4260	0.7413	15.8340
TS - T1_E vs. **T1_T**	0.5535	0.7629	0.4681	1.7393	0.3899	7.7585
AGE*TS -T2_D vs. **T1_T** ^†^	−0.4156	0.1991	0.0369	0.6599	0.4467	0.9750
AGE*TS - T1_D vs. **T1_T** ^†^	−0.4989	0.1902	0.0087	0.6072	0.4182	0.8815
AGE*TS - T1_E vs. **T1_T** ^†^	−0.2392	0.1835	0.1923	0.7873	0.5494	1.1280
DIST	0.4115	0.1155	0.0004	1.5091	1.2034	1.8925
YEAR	0.1844	0.0759	0.0151	1.2025	1.0363	1.3953
DIST*YEAR	−0.0543	0.0181	0.0027			
CMCAR	−0.1062	0.0466	0.0226	0.8992	0.8208	0.9852

^†^ Result is a ratio of odds ratios. AGE—age, years; TS—track-surface combination, T1-D, T2-D, T1-E, T1-T, T2-T; TRACK—racetrack for work-event, Track 1 (T1), Track 2 (T2); SURF—track surface, dirt (D), synthetic (E), turf (T); DIST—race or workout distance, furlongs, f; YEAR—calendar year, 0–12 (2003–2015); CMCAR—cumulative career races + workouts (divided by 10). Referents for categorical variables are in bold.

**Table 6:** Fatality rates, interval from race work-event to fatality and distribution of consolidated presenting problems for finish position groups for thoroughbred racehorse fatalities in Ontario racing from 2003–2015.

	FPOS Group									
FPOS Group ^1^	0 (137)		2.5 (51)		5.5 (51)		9 (144)		12.5 (42)	
Fatality Rate ^2^	0.5599		0.7149		1.5286		3.3148		11.2570	
Mean Interval ^3^	0.724		0.804		1.059		1.035		1.024	
IQR	0–2		0–2		0–2		0–2		0–2	
Problem	% ^4^	Rate ^5^	%	Rate	%	Rate	%	Rate	%	Rate
MSI	61.83	0.3529	50	0.3485	63.86	0.9761	77.46	2.5441	74.58	8.2583
DS	13.74	0.0784	8.97	0.0626	19.28	0.2947	8.92	0.293	18.64	2.0646
COL	7.63	0.0436	11.54	0.0804	4.82	0.0737	3.76	0.1233	0	0
MED	3.44	0.0196	7.69	0.0536	2.41	0.0368	2.82	0.0925	1.69	0.1877
IAT	2.29	0.0131	8.97	0.0626	0	0	0.94	0.0308	0	0
ACC	7.63	0.0436	8.97	0.0626	4.82	0.0737	3.29	0.1079	1.69	0.1877
NEUR	2.67	0.0152	1.28	0.0089	2.41	0.0368	2.82	0.0925	1.69	0.1877
HEM	0.76	0.0044	1.28	0.0089	2.41	0.0368	0	0	1.69	0.1877
Unknown	0	0	1.28	0.0089	0	0	0	0	0	0
	100		100		100		100		100	
Total fatalities	262		78		83		213		59	

^1^ Numbers in parentheses are group sizes from which interval data were derived-these details were not available for all horses. ^2^ Overall fatality rate for the FPOS group. ^3^ Mean interval in days from last recorded work event to death. ^4^ % distribution of presenting problem by FPOS group. ^5^ Fatality rate per 1000 group-specific race events by FPOS group. Unknown—single case with unknown presenting problem. IQR—interquartile range in days. FPOS = 0 are workouts. MSI—musculoskeletal injury; DS—died suddenly; COL—colic; MED—medical complaint; IAT—iatrogenic; ACC—accident; NEUR—neurological; HEM—hemorrhage.

## Data Availability

Mortality data are the property of the Alcohol and Gaming Commission of Ontario, Regulatory Compliance Branch, to whom requests should be directed. Thoroughbred performance data are the property of Equibase (Equibase Company LLC, 821 Corporate Drive, Lexington, KY, USA), to whom requests should be directed.

## Supplementary Materials

The following are available online at https://www.mdpi.com/article/10.3390/ani11102950/s1, Table S1. Results of logistic regression modelling of associations with fatality on Ontario racetracks, 2003–2015, for Thoroughbred race and workout work-events combined, by work-event. Table S2. Results of logistic regression modelling of associations with fatality on Ontario racetracks, 2003–2015, for Thoroughbred race work-events, by horse-year. Table S3. Results of logistic regression modelling of associations with fatality on Ontario racetracks, 2003–2015, for Thoroughbred workout work-events, by horse-year. Figure S1. Fatality rate per 1000 race events for Ontario Thoroughbred racehorses for the period 2003–2015, unit of interest—Race work-event. The graph shows fatality rate by actual finish position for horses finishing last (light blue), and the aggregate for the rest of the field (dark blue) by race field size (RSIZE). Data are raw counts and have not been controlled for any other factor. Fatality rate is high for last place finishers for all values of RSIZE and highest in races with larger field sizes, where it ranges from 3.553 to 17.268/1000. Aggregated fatality rate for the remainder of the field follows a similar pattern but ranges only from 0.396 to 1.202/1000. There were no fatalities in races of field size 3–4 (*n* = 166) or 15–17 (*n* = 4). Figure S2. Fatality rate per 1000 race events for Ontario Thoroughbred racehorses for the period 2003–2015. The graph depicts raw rates by track/surface combinations and distance (furlongs), unit of interest—Race work-event. Rates have not been controlled for the effect of any other variable. There is wide variation, but the highest fatality rates are for races above 1 mile on turf at Track 2 and composite surface at Track 1. This effect did not reach statistical significance in multivariable modelling. T2-D—Track 2 dirt; T1-D—Track 1 dirt; T1-E—Track 1 composite/all-weather; T1-T—Track 1 turf; T2-T—Track 2 turf. Figure S3. Probability of fatality (%) for workout horse-years for Ontario Thoroughbred racehorses for the period 2003–2015 and describing an age by track/surface combination interaction (AGE*TS) identified during multivariable logistic regression modelling of associations with fatality, unit of interest—event by horse-year. The pattern is very similar to that identified for the same interaction with work-event as the unit of interest (Figure 6). (Note log scale on *y*-axis.) T2-D—Track 2 dirt; T1-D—Track 1 dirt; T1-E—Track 1 synthetic; T1-T—Track 1 turf.
