# Supplementary material for: Factors Associated with Fatality in Ontario Thoroughbred Racehorses: 2003–2015
Source: Animals (Basel). 2021 Oct 13;11(10):2950. doi: 10.3390/ani11102950 (PMC8532649; doi:10.3390/ani11102950)
Supplement: Supplementary file 1 [file animals-11-02950-s001.zip › animals-1415097-supplementary/Supplementary Material Figure S1.pdf]

Supplementary Material – Figure S1.

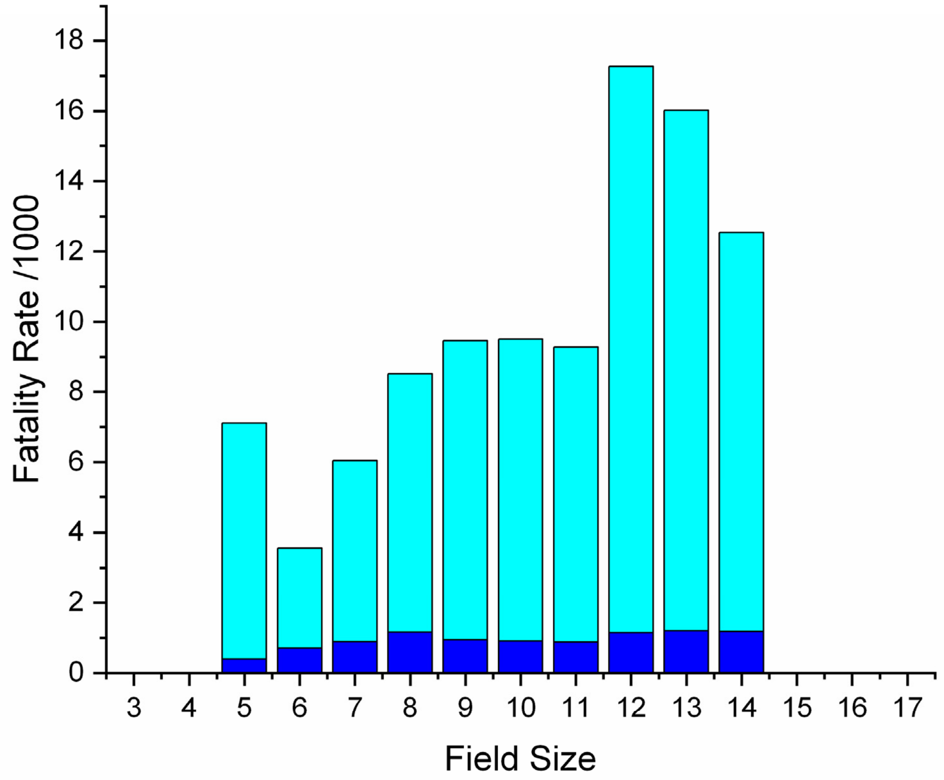

Figure S1. Fatality rate per 1000 race events for Ontario Thoroughbred racehorses for the period 2003-2015, unit of interest - race work-event. The graph shows fatality rate by actual finishing position for horses finishing last (light blue), and the aggregate for the rest of the field (dark blue) by race field size (RSIZE). Data are raw counts and have not been controlled for any other factor. Fatality rate is high for last place finishers for all values of RSIZE and highest in races with larger field sizes, where it ranges from 3.553 to 17.268/1000. Aggregated fatality rate for the remainder of the field follows a similar pattern but ranges only from 0.396 to 1.202/1000. There were no fatalities in races of field size 3-4 (n=166) or 15-17 (n=4).
