# Supplementary material for: Factors Associated with Fatality in Ontario Thoroughbred Racehorses: 2003–2015
Source: Animals (Basel). 2021 Oct 13;11(10):2950. doi: 10.3390/ani11102950 (PMC8532649; doi:10.3390/ani11102950)
Supplement: Supplementary file 1 [file animals-11-02950-s001.zip › animals-1415097-supplementary/Supplementary Material Figure S2.pdf]

Supplementary Material – Figure S2

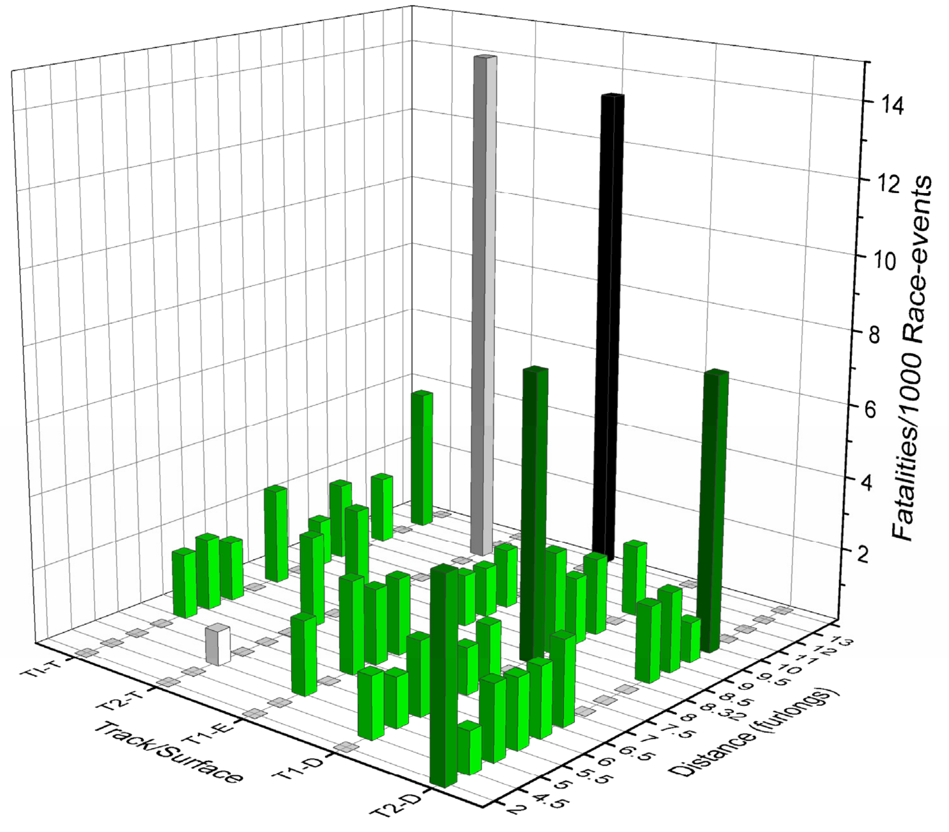

Figure S2. Fatality rate per 1000 race events for Ontario Thoroughbred racehorses for the period 2003-2015. The graph depicts raw rates by track/surface combinations and distance (furlongs), unit of interest – race work-event. Rates have not been controlled for the effect of any other variable. There is wide variation, but the highest fatality rates are for races above 1 mile on Turf at Track 2 and synthetic surface at Track 1. This effect did not reach statistical significance in multivariable modelling. T2-D - Track 2 dirt; T1-D - Track 1 dirt; T1-E - Track 1 synthetic; T1-T - Track 1 turf; T2-T - Track 2 turf.
