# Supplementary material for: Factors Associated with Fatality in Ontario Thoroughbred Racehorses: 2003–2015
Source: Animals (Basel). 2021 Oct 13;11(10):2950. doi: 10.3390/ani11102950 (PMC8532649; doi:10.3390/ani11102950)
Supplement: Supplementary file 1 [file animals-11-02950-s001.zip › animals-1415097-supplementary/Supplementary Material Figure S3.pdf]

Supplementary Material – Figure S3.

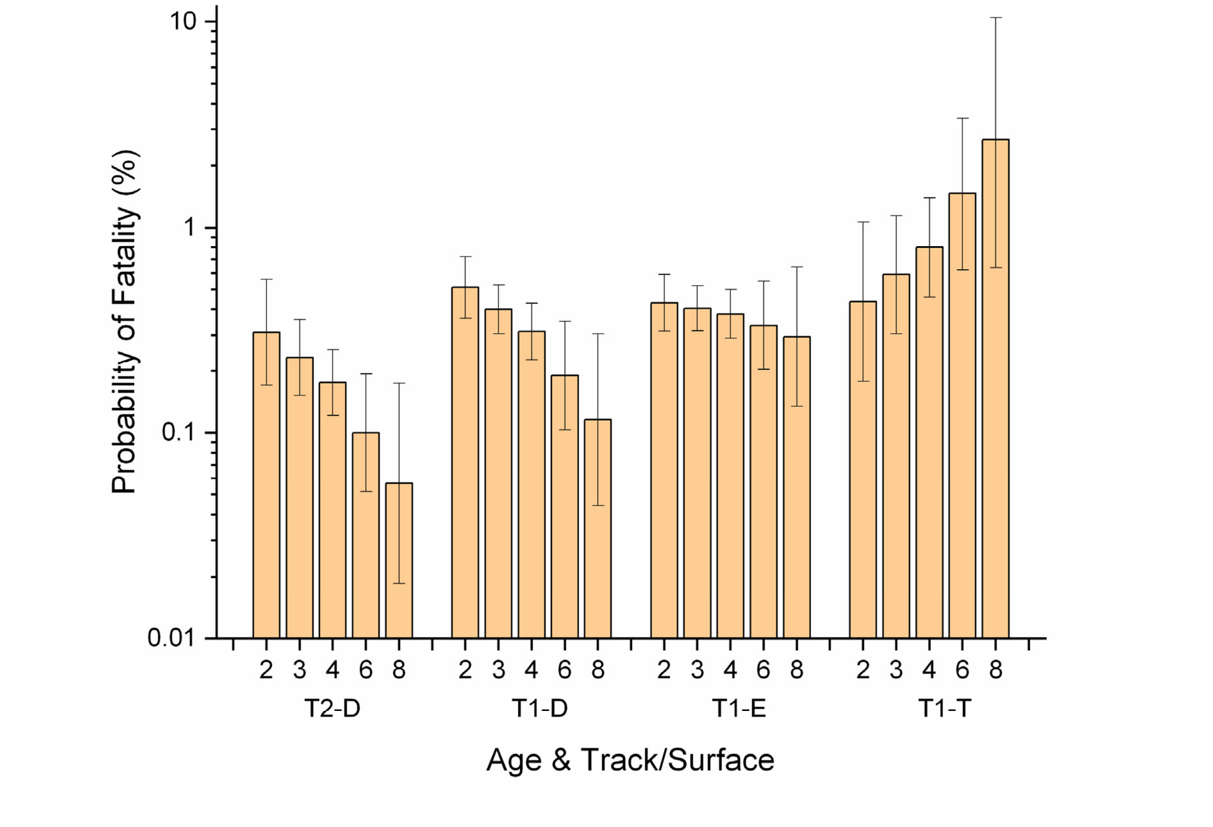

Figure S3 Probability (%) of fatality for workout horse-years for Ontario Thoroughbred racehorses for the period 2003-2015 and describing an age by track/surface combination interaction (AGE*TS) identified during multivariable logistic regression modelling of associations with fatality, unit of interest - workout event by horse-year. The pattern is very similar to that identified for the same interaction with work-event as the unit of interest (Figure 6). (Note log scale on Y-axis.) T2-D - Track 2 dirt; T1-D - Track 1 dirt; T1-E - Track 1 synthetic; T1-T - Track 1 turf.
