# Supplementary material for: Factors Associated with Fatality in Ontario Thoroughbred Racehorses: 2003–2015
Source: Animals (Basel). 2021 Oct 13;11(10):2950. doi: 10.3390/ani11102950 (PMC8532649; doi:10.3390/ani11102950)
Supplement: Supplementary file 1 [file animals-11-02950-s001.zip › animals-1415097-supplementary/Supplementary Material Table S1.pdf]

Supplementary Material – Table S1.

Table S1. Results of Logistic Regression Modelling of Associations with Fatality On Ontario Racetracks, 2003-2015, for Thoroughbred Race and Workout Work-events Combined, by Work-event,

Events	695401					
Fatalities	685				Conf. Int.	
Parameter	Estimat	s.e.m.	p-value	OR †	Lower	Upper
Intercept	-4.6408	0.2664				
AGE	0.0292	0.0484	0.5470	1.0296	0.9364	1.1320
YEAR	-0.0714	0.0305	0.0192	0.9311	0.8771	0.9884
AGE*YEAR	0.0181	0.0079	0.0213			
SEX, G vs F	-0.0178	0.0908	0.8445	0.9823	0.8222	1.1737
SEX, S vs F	0.3302	0.0995	0.0009	1.3912	1.1448	1.6907
FPOS, 0 vs. 12.5	-2.9455	0.1511	<.0001	0.0526	0.0391	0.0707
FPOS, 2.5 vs. 12.5	-2.7869	0.1754	<.0001	0.0616	0.0437	0.0869
FPOS, 5.5 vs. 12.5	-1.9963	0.1732	<.0001	0.1358	0.0967	0.1907
FPOS, 9 vs. 12.5	-1.2388	0.1495	<.0001	0.2897	0.2161	0.3884
DOWK, Fri vs. Wed	-0.1832	0.1642	0.2646	0.8326	0.6035	1.1487
DOWK, Sat vs. Wed	0.0393	0.1464	0.7885	1.0401	0.7806	1.3857
DOWK, Sun vs. Wed	0.1298	0.1428	0.3633	1.1386	0.8606	1.5063
DOWK, Mon vs. Wed	0.2260	0.1675	0.1774	1.2536	0.9028	1.7407
DOWK, Tues vs. Wed	-0.0882	0.1931	0.6478	0.9156	0.6271	1.3368
DOWK, Thurs vs. Wed	0.4406	0.1622	0.0066	1.5536	1.1305	2.1351
TRACK, T2 vs. T1	-0.0720	0.1799	0.6890	0.9305	0.6540	1.3239
CMD (n/10)	0.0243	0.0070	0.0006	1.0245	1.0105	1.0388
CMD*TRACK, T2 vs. T1 †	0.0304	0.0138	0.0278	1.0308	1.0033	1.0591
CMCAR (n/10)	-0.1515	0.0282	<.0001	0.8594	0.8133	0.9082

† Result is a ratio of odds ratios. AGE - age, years; SEX - sex, F-female, G-gelding, S-stallion; DOWK - day of week for work-event; TRACK - racetrack for work-event, Track 1 (T1), Track 2 (T2); FPOS - race event finish position group, 2.5 (1-4), 5.5 (5, 6), 9 (7-11), 12.5 (>11); YEAR - calendar year, 0-12 (2003-2015); CMCAR - cumulative career races + workouts (divided by 10); CMD - cumulative days in work from first to current work-event (divided by 10). Referents for categorical variables are underlined.
