# Supplementary material for: Factors Associated with Fatality in Ontario Thoroughbred Racehorses: 2003–2015
Source: Animals (Basel). 2021 Oct 13;11(10):2950. doi: 10.3390/ani11102950 (PMC8532649; doi:10.3390/ani11102950)
Supplement: Supplementary file 1 [file animals-11-02950-s001.zip › animals-1415097-supplementary/Supplementary Material Table S2.pdf]

Supplementary Material – Table S2.

Table S2. Results of Logistic Regression Modelling of Associations with Fatality On Ontario Racetracks, 2003-2015, for Thoroughbred Race Work-events, by Horse Year

Race Horse Years	44639					
Mortalities	433					
Parameter	Estimat	Error	p-value	OR	L-OR	U-OR
Intercept	2.0681	2.0510				
TRACK, T2 vs. **T1**	-0.3457	0.1173	0.0032	0.7077	0.5624	0.8907
SEX, G vs **F**	-0.3244	0.2949	0.2713	0.7230	0.4056	1.2887
SEX, S vs. **F**	0.0940	0.3852	0.8072	1.0985	0.5163	2.3372
FPOS, 2.5 vs. **12.5**	-7.3493	2.0612	0.0004	0.0006	0.00001	0.0365
FPOS, 5.5 vs. **12.5**	-4.5572	2.0633	0.0272	0.0105	0.0002	0.5986
FPOS, 9 vs. **12.5**	-3.3522	2.0449	0.1012	0.0350	0.0006	1.9267
FPOS*SEX, 2.5/G vs. **12.5/F**	1.1178	0.3953	0.0047	3.0581	1.4092	6.6365
FPOS*SEX, 2.5/S vs. **12.5/F**	0.6014	0.5091	0.2375	1.8247	0.6727	4.9492
FPOS*SEX, 5.5/G vs. **12.5/F**	-0.1330	0.3908	0.7335	0.8755	0.4070	1.8832
FPOS*SEX, 5.5/S vs. **12.5/F**	-0.0673	0.4980	0.8926	0.9350	0.3523	2.4814
FPOS*SEX, 9/G vs. **12.5/F**	0.4596	0.3318	0.1660	1.5834	0.8264	3.0341
FPOS*SEX 9/S vs. **12.5/F**	0.2572	0.4337	0.5531	1.2933	0.5527	3.0260
YEAR	-0.0308	0.0138	0.0259	0.9696	0.9437	0.9963
RWYN	0.2281	0.0724	0.0016	1.2562	1.0900	1.4477
RWYN*RWYN	-0.0140	0.0053	0.0077			
YD	-0.1555	0.0487	0.0014	0.8560	0.7781	0.9417
CMD	0.1758	0.0427	<.0001	1.1922	1.0964	1.2963
YD*CMD	-0.0183	0.0042	<.0001			
RSIZE	-0.3435	0.1586	0.0303	0.7093	0.5198	0.9679
RSIZE*FPOS, 2.5 vs. **12.5** †	0.4021	0.1652	0.0149	1.4950	1.0815	2.0666
RSIZE*FPOS, 5.5 vs., **12.5** †	0.1770	0.1690	0.295	1.1936	0.8571	1.6624
RSIZE*FPOS, 9 vs. **12.5** †	0.1010	0.1634	0.5364	1.1063	0.8031	1.5239

† Result is a ratio of odds ratios. SEX - sex, F-female, G-gelding, S-stallion; RSIZE - race field size; TRACK - racetrack for work-event, Track 1 (T1), Track 2 (T2); FPOS - race event finish position group, 2.5 (1-4), 5.5 (5, 6), 9 (7-11), 12.5 (>11); YD - yearday, 1-365 (divided by 30); YEAR - calendar year, 0-12 (2003-2015); CMD - cumulative days in work from first to current work-event (divided by 10); RWYN - total races or workouts in season to current work-event. Referents for categorical variables are in bold.
