# Supplementary material for: Factors Associated with Fatality in Ontario Thoroughbred Racehorses: 2003–2015
Source: Animals (Basel). 2021 Oct 13;11(10):2950. doi: 10.3390/ani11102950 (PMC8532649; doi:10.3390/ani11102950)
Supplement: Supplementary file 1 [file animals-11-02950-s001.zip › animals-1415097-supplementary/Supplementary Material Table S3.pdf]

Supplementary Material – Table S3

Table S3. Results of Logistic Regression Modelling of Associations with Fatality On Ontario Racetracks, 2003-2015, for Thoroughbred Workout Work-events, by horse year.

Workout Horse Years	52658					
Fatalities	251				Conf. Int.	
Parameter	Estimate	Error	p-value	OR	Lower	Upper
Intercept	-4.3914	0.9811				
DOWK, Fri vs. **Wed**	-0.1432	0.2644	0.5881	0.8666	0.5161	1.4550
DOWK, Mon vs. **Wed**	0.3753	0.2492	0.1320	1.4554	0.8930	2.3720
DOWK, Sat vs. **Wed**	0.0226	0.2407	0.9253	1.0228	0.6381	1.6394
DOWK, Sun vs. **Wed**	0.0808	0.2367	0.7330	1.0841	0.6817	1.7241
DOWK, Thu vs. **Wed**	0.4933	0.2561	0.0541	1.6377	0.9914	2.7054
DOWK, Tue vs. **Wed**	-0.2748	0.3211	0.3921	0.7597	0.4049	1.4255
SURF, T2-D vs. **T1-T**	0.8310	0.9217	0.3673	2.2956	0.3770	13.9790
SURF, T1-D vs. **T1-T**	1.2671	0.8211	0.1228	3.5505	0.7102	17.7510
SURF, T1-E vs. **T1-T**	0.7274	0.8044	0.3659	2.0697	0.4277	10.0140
AGE	0.3055	0.1733	0.0779	1.3573	0.9664	1.9063
SEX, G vs **F**	0.2067	0.1688	0.2208	1.2296	0.8832	1.7118
SEX, S vs. **F**	0.4463	0.1480	0.0026	1.5625	1.1691	2.0884
RWYN	0.1442	0.0531	0.0066	1.1551	1.0410	1.2817
CMYR	-1.3385	0.4352	0.0021	0.2622	0.1117	0.6154
YD	0.0171	0.1767	0.9229			
YD*YD	-0.0256	0.0126	0.0413			
AGE*SURF, T2-D vs. **T1-T** †	-0.5878	0.2153	0.0063	0.5555	0.3643	0.8472
AGE*SURF, T1-D vs. **T1-T** †	-0.5530	0.1977	0.0051	0.5752	0.3904	0.8475
AGE*SURF, T1-E vs. **T1-T** †	-0.3692	0.1900	0.0520	0.6913	0.4764	1.0032

† Result is a ratio of odds ratios. AGE - age, years; SEX - sex, F-female, G-gelding, S-stallion; DOWK - day of week for work-event; SURF - track surface, dirt (D), synthetic (E), turf (T); YD - yearday, 1-365 (divided by 30); CMYR - total races + workouts in season to current work-event (divided by 10); RWYN - total races OR workouts in season to current work-event. Referents for categorical variables are in bold.
